# Radiation dose escalation for locally advanced nasopharyngeal carcinoma patients with local and/or regional residual lesions after standard chemoradiotherapy: a non-randomized, observational study

**DOI:** 10.1186/s13014-022-02147-7

**Published:** 2022-11-07

**Authors:** Ting Jin, Nan-Fang Liu, Qi-Feng Jin, Yong-Hong Hua, Xiao-Zhong Chen

**Affiliations:** 1 The Cancer Hospital of the University of Chinese Academy of Sciences (Zhejiang Cancer Hospital), Institute of Basic Medicine and Cancer (IBMC), Chinese Academy of Sciences, Hangzhou, 310022 Zhejiang China; 2 Key Laboratory of Head and Neck Cancer Translational Research of Zhejiang Province, Hangzhou, 310022 China; 3 Department of Gynecologic Radiation Oncology, Zhejiang Cancer Hospital, Hangzhou, 310022 Zhejiang China

**Keywords:** Nasopharyngeal carcinoma, Boost, Radiotherapy, Residual lesion, Chemotherapy

## Abstract

**Background:**

To assess the effectiveness and toxicity of radiation dose escalation for locally advanced nasopharyngeal carcinoma (LA-NPC) in patients with local and/or regional residual lesion(s) after standard treatment.

**Methods:**

From November 2011 to November 2020, 259 LA-NPC patients who had local and/or regional residual lesion(s) after induction chemotherapy followed by concurrent chemoradiotherapy (IC + CCRT) from our hospital were included. The total dose of primary radiotherapy (RT) was 68.1–74.25 Gy (median, 70.4 Gy). The boost doses were 4.0–18.0 Gy (median, 9 Gy), 1.8–2.0 Gy/fraction.

**Results:**

For all patients, the 5-year local relapse-free survival was 90.2%, regional relapse-free survival was 89.1%, locoregional relapse-free survival (LRRFS) was 79.5%, distant metastasis-free survival (DMFS) was 87.9%, failure-free survival (FFS) was 69.0%, and overall survival (OS) was 86.3%. LRRFS, DMFS, FFS, and OS in patients with age ≤ 65 versus > 65, plasma Epstein-Barr virus-deoxyribonucleic acid  ≤ 500 versus > 500, T_1–2_ versus T_3–4_, N_0–1_ versus N_2–3_, and stage III versus stage IV showed no statistically significant differences. The interval between primary RT and boost was not a prognostic factor for LRRFS, DMFS, FFS, and OS. Males had a lower 3-year FFS rate than females (72.9% vs. 83.7%, P = 0.024). LA-NPCs with locally and regionally residual lesion(s) had the worst 3-year DMFS and OS rates compared with locally or regionally residual lesion(s) (77.7% vs. 98.8% vs. 87.4%, P = 0.014; 75.9% vs. 94.5% vs. 82.4%, P = 0.002).

**Conclusion:**

Boost radiation was an option for LA-NPCs with locally and/or regionally residual lesions after receiving IC + CCRT. It warrants further prospective study.

*Trial registration*: Retrospectively registered.

## Background

In 2020, 133,354 patients were newly diagnosed as nasopharyngeal carcinoma (NPC) worldwide, and 80,008 related deaths were reported [1]. Approximately 50% of all NPC patients worldwide reside in China [2, 3]. Approximately two-thirds of patients are stage III–IVA at the initial diagnosis [4]. Induction chemotherapy (IC) followed by concurrent chemoradiotherapy (CCRT) is recommended as one of the standard management options for stage III–IVA NPC patients (except T3N0, category 1) [5].

Despite the widespread use of intensity-modulated radiotherapy (IMRT), 16.7-40.1% of patients at stage III–IVA NPC did not achieve complete response (CR) at the end of radiotherapy (RT), as assessed by magnetic resonance imaging (MRI) [6, 7]. This proportion was 25.6% when assessed three months after RT [8]. MRI-detected residual lesion(s) at the end of or three months after RT is associated with a poor outcome. On MRI at the end of RT, patients without residual lesion(s) had a 3-year overall survival (OS) rate of 90% compared to 73% in patients with these lesions (P = 0.007); the local relapse-free survival (LRFS) rate was 97% versus 89% (P = 0.002), and disease-free survival (DFS) rate was 82% versus 67% (P = 0.001) [7]. Similarly, on MRI 3 months after RT, the 5-year OS rate was 93.8% in patients without residual lesion(s) versus 76.6% (P < 0.001) in those with these lesions, progression-free survival (PFS) rate was 84.7% versus 67.9% (P = 0.006), the LRFS rate was 93.4% versus. 80.4% (P = 0.002), and the distant metastasis-free survival (DMFS) rate was 90.3% versus 87.9% (P = 0.305) [8].

To improve the prognosis of patients with residual lesion(s), boost RT has been commonly employed for patients who have residual lesion(s) after RT in our hospital. This study assessed the long-term efficacy and toxicity of boost RT for LA-NPC in patients with local and/or regional residual lesion(s) who previously received IC + CCRT.

## Methods

### Patients

This is a non-randomized, observational study. The inclusion criteria were (1) histopathologically confirmed NPC (WHO type II/III); (2) patients who were 18–70 years old; (3) patients with 8th American Joint Committee on Cancer stage III–IVA NPC who had completed IC + CCRT; (4) residual tumors located in the nasopharynx (persistent tumor mass or thickened nasopharyngeal walls), soft tissues (low signal in T1, high signal in T2, and enhancement following the administration of gadolinium diethylenetriamine pentaacetic acid), skull base, regional lymph nodes (the diameter of the short axis of the neck lymph node was greater than 10 mm while retropharyngeal lymph node greater than 5 mm) assessed by nasopharyngoscopy and/or MRI. Specific diagnostic criteria are have been described previously [7, 9, 10]. The exclusion criteria were (1) patients with a previous malignant tumor within five years; (2) copresence of a second primary tumor; (3) pregnancy or lactation; and (4) distant metastasis during treatment.

### Chemotherapy and RT

IC regimens included paclitaxel + cisplatin + fluorouracil, gemcitabine + cisplatin, paclitaxel + cisplatin, or cisplatin + fluorouracil, which were repeated every three weeks. Cisplatin, 80–100 mg/m^2^ every three weeks or 25–40 mg/m^2^ every week, was the concurrent chemotherapy regimen.

RT was performed using IMRT, volumetric modulated arc therapy, or helical tomotherapy. Reports No. 50 and No. 62 of the International Commission on Radiation Units and Measurements (ICRU) were used to determine target volumes. Enhanced MRI was used as a reference in the delineation of the target. The gross tumor volume for the nasopharynx and retropharyngeal lymph nodes (GTVnx + rn) contained the primary tumor and positive retropharyngeal lymph nodes. The metastatic lymph node gross tumor volume (GTVnd) included positive cervical lymph nodes. The clinical target volume (CTV)1 was the GTVnx + rn plus a 5–10 mm margin. CTV2 was a 5–10 mm expansion from CTV1 plus high-risk regions based on the tumor invasion pattern. Extending the GTV or CTV by 3 mm yielded corresponding planning target volumes (PTVs). The prescribed doses for the planning gross target volume of the nasopharyngeal and retropharyngeal lymph node (PGTVnx + rn) was 69.96–70.4 Gy/32–33 fractions; planning gross target volume of the cervical lymph nodes (PGTVnd), 66–70.4 Gy/32–33 fractions; PTV1, 60.8–61.05 Gy/32–33 fractions, and PTV2, 54.4–54.45 Gy/32–33 fractions. Critical tissue dose limitation and plan assessment referred to IMRT target volume and dose design guideline for NPC [11].

IMRT boost irradiation was administered to patients who have detectable locally and/or regionally residual tumors. Extending GTV-r by 3 mm yielded the PGTV-r. The total boost doses to the local and/or regional residual lesion(s) were 4.0–18.0 Gy (median, 9 Gy) and 1.8–2.0 Gy/fraction.

### Univariate analysis

Univariate analyses included demographic (gender, age) and clinical data (plasma EBV-DNA, T category, N category, stage, residual sites, time to boost after primary RT).

### Statistical analysis

The endpoints included LRFS, regional relapse-free survival (RRFS), locoregional relapse-free survival (LRRFS), DMFS, failure-free survival (FFS), and OS. All the endpoints were defined as the interval from the date of initiation of treatment to the date of the failure or the last follow-up. Toxicity criteria of the Radiation Therapy Oncology Group were used to assess radiation-related toxic effects [12].

The time-to-event endpoints analysis was conducted using the Kaplan–Meier method, and differences between the groups were analyzed using the log-rank test. The *χ*2 tests were used to compare categorical variables. SPSS version 21.0 (IBM Corp., Armonk, NY, USA) was used in this study. All statistical tests were two-sided, and statistical significance was defined as a P < 0.05.

## Results

### Patient characteristics

From November 2011 to November 2020, 259 LA-NPC patients who had a residual local and/or regional lesion after receiving IC + CCRT from our hospital were included. Table 1 lists the patient characteristics. The ratio of males to females was close to 3:1. Among the 259 patients included in this study, the cervical lymph node was the most common site for residual lesion(s) (58.7%), followed by the primary focus (46.7%), and the retropharyngeal lymph node was the least common site (20.8%). The last follow-up was May 14, 2021 (Table 2).

**Table 1:** Clinical characteristics of study participants

Characteristic	No. patients		Percentage
Total	259		100
Sex			
Male	194		74.9
Female	65		25.1
Median age (range); years	49 (19–74)		
T category			
T 1–2	34		13.1
T 3–4	225		86.9
N category			
N 0–1	89		34.4
N 2–3	170		65.6
Stage			
III	129		49.8
IVA	130		50.2
Plasma EBV-DNA (copies/mL)			
≤ 500	173		66.8
> 500	86		33.2
95% of target volume received dose greater than (D95, Gy)			
Average dose of nasopharynx (PTVnx + rn) (range)	70.2 (66.1–74.3)		
Average dose of neck node (PTVnd) (range)	69.4 (60.7–75.9)		
Sites of residual			
Primary focus	121	46.7	
Retropharyngeal lymph nodes	54	20.8	
Cervical lymph nodes	152	58.7	
Median time to boost after RT (range); days	36 (1–74)		
Average boost dose for primary focus (range); Gy	8.1 (4–12.5)		
Average boost dose for retropharyngeal lymph nodes (range); Gy	8.3 (5–12.5)		
Average boost dose for cervical lymph nodes (range); Gy	8.6 (4–18)		

*EBV-DNA* Epstein-Barr virus-deoxyribonucleic acid, *PTVnx + rn* planning gross target volume of the nasopharyngeal and retropharyngeal lymph node, *PTVnd* planning gross target volume of the cervical lymph nodes, *RT* radiotherapy

**Table 2:** Clinical characteristics of study participants

Prognostic factor	3-year LRRFS	*χ*^2^	*P*^a^	3-year DMFS	*χ*^2^	*P*^a^	3-year FFS	*χ*^2^	*P*^a^	3-year OS	*χ*^2^	*P*^a^
Gender		2.977	0.084		2.245	0.134		5.094	**0.024**		1.091	0.296
Male	82.9			88.5			72.9			89.5		
Female	89.3			94.2			83.7			96.3		
Age		1.414	0.234		0.209	0.648		0.544	0.461		2.276	0.131
≤ 65	85.6			89.7			76.4			91.7		
> 65	72.3			94.7			68.2			84.4		
Plasma EBV-DNA (copies/mL)		0.384	0.536		0.001	0.976		0.124	0.725		0.776	0.378
≤ 500	83.0			90.6			75.3			90.0		
> 500	87.7			88.5			76.4			93.6		
T category		0.013	0.911		0.707	0.401		0.587	0.444		0.069	0.793
T 1–2	82.0			93.1			78.7			91.0		
T 3–4	85.2			89.4			75.3			91.4		
N category		1.449	0.229		0.235	0.628		1.994	0.158		0.226	0.634
N 0–1	87.9			90.2			79.5			92.3		
N 2–3	83.0			89.9			73.8			90.7		
Stage		0.238	0.626		2.024	0.155		1.922	0.166		1.184	0.277
III	85.7			91.4			78.2			93.3		
IVA	83.3			88.7			73.2			88.9		
Time to boost after radiotherapy (days)		1.077	0.584		1.749	0.417		0.902	0.637		0.432	0.806
1–14	92.0			81.6			73.8			90.0		
15–28	88.8			90.5			79.7			95.4		
> 28	81.2			92.4			75.0			90.2		
Sites of residual		3.533	0.171		8.580	**0.014**		4.181	0.124		12.631	**0.002**
Primary focus	79.4			98.8			78.2			94.5		
Lymph nodes	90.2			87.4			78.4			92.4		
Primary focus + lymph nodes	76.2			77.7			57.6			75.9		

Statistically significant value is shown in bold

*DMFS* distant metastasis-free survival, *EBV-DNA* Epstein-Barr virus-deoxyribonucleic acid, *FFS* failure-free survival, *LRRF* locoregional relapse-free survival, *OS* overall survival

^a^Log-rank test

### Antitumor activity

**Fig. 1:**
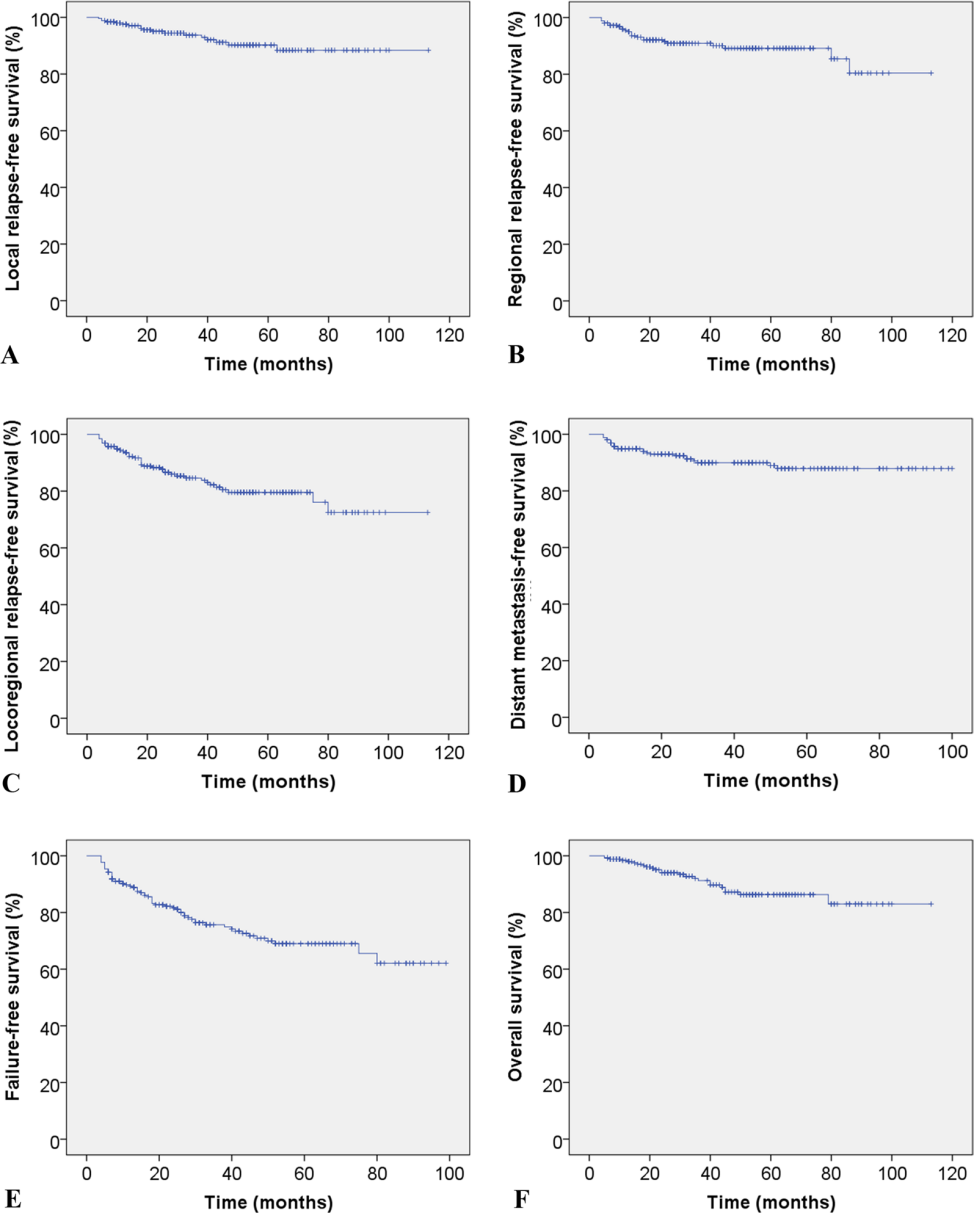
Kaplan–Meier estimates of **A** local relapse-free survival for all patients. **B** Regional relapse-free survival for all patients. **C** Locoregional relapse-free survival for all patients. **D** Distant metastasis-free survival for all patients. **E** Failure-free survival for all patients. **F** Overall survival for all patients

The median interval between primary radical RT and boost RT was 36 (1–74) days. The average boost dose for primary focus, retropharyngeal lymph nodes, and cervical lymph nodes was 8.1 (4–12.5) Gy, 8.3 (5–12.5) Gy, and 8.6 (4–18) Gy, respectively. The median follow-up was 41 (range 5–113) months. The 3- and 5-year LRFS rates were 93.7% and 90.9%; those for RRFS were 90.9% and 89.1%; those for LRRFS were 84.6% and 79.5%; those for DMFS were 89.9% and 87.9%; those for FFS were 75.7% and 69.0%; and those for OS were 91.2% and 86.3%, respectively. Figure 1 shows survival curves for different endpoints.

### Univariate analysis

LRRFS, DMFS, FFS, and OS in patients with age ≤ 65 versus > 65 years, plasma EBV-DNA ≤ 500 versus > 500 copy number, T_1–2_ versus T_3–4_, N_0–1_ versus N_2–3_, and stage III versus stage IV had no statistically significant differences. The interval between primary and boost RT was not a prognostic factor for LRRFS, DMFS, FFS, and OS. Male patients had a lower three-year FFS rate than female patients (72.9% vs. 83.7%, P = 0.024). Patients with local and regional residual lesion(s) had the worst 3-year DMFS and OS rates compared with those with only locally or only regionally residual lesions (77.7% vs. 98.8% vs. 87.4%, P = 0.014; 75.9% vs. 94.5% vs. 82.4%, P = 0.002).

### Adverse events

The addition of boost RT to primary RT was well tolerated. Dry mouth (77.6%), followed by neck tissue damage (47.1%) and ear (deafness/otitis) (34.7%) were the most common radiation-related late adverse events of any grade. Ear (deafness/otitis) (10.0%), followed by dry mouth (7.7%) and neck tissue damage (1.9%) were the most common grade 3–4 radiation-related late adverse events.

Four patients developed grade 3–4 cranial neuropathy, and three of them required long-term enteral nutrition via a percutaneous endoscopic gastrostomy (PEG). In total, 9.7% and 1.2% patients experienced grades 1–4 and 3–4 symptomatic temporal lobe necrosis. Table 3 lists the details of the side effects in the boost RT group.

**Table 3:** Late radiotherapy-related toxic effects

Toxicity	Any grade	Grade 3–4
	No. of patients (%)	No. of patients (%)
Symptomatic temporal lobe necrosis	25 (9.7)	3 (1.2)
Cranial neuropathy	24 (9.3)	4 (1.5)
Eye damage	4 (1.5)	2 (0.8)
Ear (deafness/otitis)	90 (34.7)	26 (10.0)
Bone necrosis	10 (3.8)	2 (0.8)
Trismus	19 (7.3)	2 (0.8)
Dry mouth	201 (77.6)	20 (7.7)
Neck tissue damage	122 (47.1)	5 (1.9)

## Discussion

The present study is the first large, single-arm study to assess the efficacy and toxicity of radiation dose escalation for LA-NPC in patients with local and/or regional residual lesion(s) after they received IC + CCRT. Our study showed high 5-year rates in all patients who received boost irradiation: LRFS, 90.2%; RRFS, 89.1%; LRRFS, 79.5%; DMFS, 87.9%; FFS, 69.0%; and OS, 86.3%.

Biopsy and histopathology are the gold standard for detecting residual lesion(s). A pathological examination should be performed when residual lesions are suspected, if conditions permit. In the present study, patients who were suspected to have residual lesions via electronic nasopharyngoscopy routinely underwent a biopsy. When MRI indicated residual lesion(s) in cervical lymph nodes after RT, an ultrasound-guided fine-needle puncture was routinely used to confirm whether live tumor cells were present. However, most residual lesions at the primary site of LA-NPC are located in the deep tissue, such as the skull base, parapharyngeal space, intracranial area, and paranasal sinuses, where biopsy cannot be performed. In such cases, enhanced MRI was performed to determine the presence of the above residual tumors.

Selecting the best time node is crucial to evaluate residual tumor(s). The advantage of immediately evaluating for a residual tumor at the end of RT is that immediate treatment can be initiated that can improve the curative effect. The disadvantage is that initiating evaluation for residual tumor(s) at the end of RT may be associated with a certain false-positive rate. In such cases, over-treatment may occur, which may lead to increase in toxicity and adverse effects. The advantage of delayed evaluation is that overtreatment can be avoided in false-positive patients, but the disadvantage is that treatment may be delayed in true-positive patients resulting in poor prognosis. The best time for evaluation for residual lesion(s) is three months after RT in patients with NPC who previously have received conventional two-dimensional radical RT because nearly 80% residual lesion(s) subside spontaneously within three months after RT [13].

Currently, IMRT followed by IC has become the standard management for LA-NPC. The regression mode of NPC has remarkably changed. Previous studies found that about 10%-22% patients achieved CR after IC [6, 14, 15]. Our previous study found that about 90% patients achieved CR after IC + CCRT, which is much higher than that for patients who received RT alone or CCRT [7, 8, 16, 17].

In two retrospective studies [7, 18] in patients with LA-NPC on the prognostic value of MRI-detected residual lesion(s) immediately after IMRT, patients with residual tumor(s) following IMRT had a worse prognosis than patients without residual lesion(s). Lv et al. [8] assessed the prognostic value of residual lesion(s) detected by MRI three months after IMRT in 664 NPC patients and found that patients without residual lesion(s) three months after IMRT had a better prognosis than those with MRI-detectable residual tumor(s) (5-year OS: 93.8% vs. 76.6%, P < 0.001; 5-year LRRFS: 93.4% vs. 80.4%, P = 0.002; 5-year PFS: 84.7% vs. 67.9%, P = 0.006; 5-year DMFS: 90.3% vs. 87.9%, P = 0.305). Although 86.4% patients (28.3% at stage I or II) received chemotherapy, the proportion of patients receiving IC was not specified. To investigate the relationship between tumor regression and prognosis, Wenfeng Li et al. [19] retrospectively conducted a study of 556 NPC patients. At 3–4 months after IMRT, patients with a clinical complete response (cCR) had a greater local–regional control rate than patients without a cCR (92.9% vs. 73.1%, P < 0.001). The same phenomenon was observed 6–9 months after IMRT (92.9% vs. 54.2%, P < 0.001). The authors also noted that early (3–4 month) and delayed (6–9 month) cCR had better outcomes compared with those without cCR (5-year OS: 92.1% vs. 90.6% vs. 65.4%, P < 0.001; 5-year LRRFS: 92.6% vs. 93.3% vs. 54.2%, P < 0.001; 5-year FFS: 83.8% vs. 84.4% vs. 48.5%, P < 0.001). The percentage of patients who received IC was not specified, and 25.7% patients had stage I or II diseases. Wang-Zhong Li et al. [20] retrospectively evaluated the predictive value of residual retropharyngeal lymph node(s) detected by MRI three months after IMRT in 1,103 NPC patients. The retropharyngeal lymph node area had residual lesion(s) in 28.2% patients. Their findings demonstrated that patients with residual lesions in the retropharyngeal lymph node(s) had worse outcomes than those who did not have residual lesions in the retropharyngeal lymph node (3-year OS: 89.5% vs. 95.0%, P < 0.001; 3-year LRRFS: 93.3% vs. 96.9%, P < 0.001; 3-year PFS: 78.4% vs. 90.4%, P < 0.001; 3-year DMFS: 83.6% vs. 94.7%, P < 0.001). Only 54.3% patients received IC and 10.4% patients had stage I or II diseases. Liu et al. [21] conducted a retrospective study of 82 NPC patients to investigate the prognosis of patients who had MRI-detected residual cervical lymphadenopathy three months after radiation. Based on the postoperative pathology of cervical lymph node dissection, 83% (62/82) patients with MRI-detected residual cervical lymphadenopathy were diagnosed with residual tumor. Besides, the authors found that in half of the patients, tumors had progressed, and the prognosis of patients without tumor cells in cervical lymph nodes was better than that of those with tumor cells in cervical lymph nodes (3-year OS: 100% vs. 83.2%, P = 0.005; P = 0.014; 3-year PFS: 83.3% vs. 49.9%, P = 0.008; 3-year RRFS: 100% vs.73.0%, 3-year LRRFS: 91.7% vs. 53.9%, P = 0.005).

Studies on whether local–regional residual tumors should be treated with boost irradiation have bene performed. In the He et al. [7] study described above the prognosis of patients with radiation boost (dose > 73.92 Gy) was not better than that of patients without radiation boost (3-year OS: 83% vs. 85%, P > 0.05; 3-year LRRFS: 93% vs. 94%, P > 0.05; 3-year DFS 76% vs. 75%, P > 0.05). Radiation boost was only administered to patients with large residual tumors and two-thirds of patients without RT boost had no residual tumor after RT. Thus, the prognosis of patients in the RT boost arm may have been significantly worse than that of patients in the non-RT boost arm. Ou et al. [22] retrospectively conducted a study of 553 patients with LA-NPC to assess the prognostic value of residual tumors based on clinical and radiologic examination immediately after IMRT. In total, 87.5% patients received IC and 13.4% had residual lesion(s) at the end of RT. Local residual diseases were treated with a boost of 2.2–4.4 Gy, once or twice a day by small-field IMRT or 8–16 Gy, once or twice a week by intracavitary afterloading treatment. Palpable residual cervical nodes were treated with a boost of 4–6 Gy in two or three fractions a day by electron field. The authors found that the prognosis of patients with radiation boost (prescribed dose > 73.92 Gy) was even worse than that of patients without radiation boost (5-year LRFS: 73.7% vs. 89.5%, P = 0.004; 5-year RRFS: 83.1% vs. 93.8%, P < 0.001; 5-year DFS 52.2% vs. 71.1%, P = 0.004). Patients without radiation boost had no residual diseases, indicating that the prognosis of patients in the radiation boost arm was significantly worse than that of patients in the non-radiation boost arm. In the Liang et al. [18] study described above, 51.9% (206/397) patients had a residual tumor(s) immediately after IMRT; 21.4% (44/206) patients received boost irradiation. The results indicated that, in patients with MRI-detected residual tumors, the outcomes of patients with radiation boost were better than that of those who did not receive radiation boost (5-year LRRFS: 95.3% vs. 83%, P = 0.034). In the present study, adding boost RT to primary RT was an effective treatment for patients with local and/or regional residual lesion(s) after receiving IC + CCRT, which is consistent with Liang et al.’s study results [18] but different from the results of He et al.’s and Ou *et* *al*’s study [7, 22]. The main reasons for these differences could be the different proportions of patients with LA-NPC and those receiving IC, as well as the timing of and criteria for residual tumor evaluation.

Although the incidence of late radiotherapy-related toxic effects was similar compared to that reported in a previous study in which patients only received IC followed by CCRT [23], our study revealed that boost RT increased the grade 3–4 symptomatic temporal lobe necrosis, followed by grade 1–2 cranial neuropathy, grade 3–4 eye damage, grade 3–4 hearing impairment, grade 3–4 dry mouth, and grade 3–4 neck tissue damage. Two (0.8%) patients developed radiative nasopharyngeal necrosis, and symptoms were alleviated after endoscopic debridement and local or systemic antibiotic treatment.

The current study has several limitations: First, this is a retrospective study and has limitations inherent to such studies; second, most patients did not receive boost RT immediately after RT; last, the boost RT doses were not uniform.

Nonetheless, our study is noteworthy since this is the first large-scale, real-world study to show that adding boost RT with primary RT is an option for LA-NPC in patients with local and/or regional residual lesions after receiving IC + CCRT. Future clinical trials should focus on appropriate patient selection, appropriate criteria for residual lesion evaluation (such as biopsy, fine-needle aspiration, plasma EBV DNA, and positron emission tomography-computed tomography/positron emission tomography-MRI scan), appropriate timing of boost RT, optimal boost RT irradiation dose selection, biomarker identification, as well as the optimal drugs in combination with boost RT that can be used to overcome RT resistance.

## Conclusion

The present study is the first large, single-arm study to indicates that boost radiation is an option for LA-NPC patients with local and/or regional residual lesions who have previously received IC + CCRT. However, a further prospective study is warranted to confirm these results.

## Data Availability

The datasets used and/or analyzed during the current study are available from the corresponding author upon reasonable request.

## Abbreviations

CR: Clinical complete response
CTV: Clinical target volume
DFS: Disease-free survival
DMFS: Distant metastasis-free survival
EBV-DNA: Epstein-Barr virus-Deoxyribonucleic acid
FFS: Failure-free survival
GTV: Gross tumor volume
GTVnx + rn: Gross tumor volume for the nasopharynx and retropharyngeal lymph nodes
GTVnd: Metastatic lymph node gross tumor volume
ICRU: Reports No. 50 and No. 62 of the International Commission on Radiation Units and Measurements
IC + CCRT: Induction chemotherapy followed by concurrent chemoradiotherapy
IMRT: Intensity-modulated radiotherapy
LA-NPC: Locally advanced nasopharyngeal carcinoma
LRFS: Local relapse-free survival
LRRFS: Locoregional relapse-free survival
MRI: Magnetic resonance imaging
NPC: Nasopharyngeal carcinoma
OS: Overall survival
PFS: Progression-free survival
PGTV: Planning gross tumor volume
PGTVnd: Planning gross target volume of the cervical lymph nodes
PTV: Planning target volume
RRFS: Regional relapse-free survival
RT: Radiotherapy
RTOG: Radiation Therapy Oncology Group
